# Seasonal and Spatial Variability of Virioplanktonic Abundance in Haihe River, China

**DOI:** 10.1155/2013/526362

**Published:** 2013-06-17

**Authors:** Lili Ma, Rui Sun, Guannan Mao, Hui Yu, Yingying Wang

**Affiliations:** Key Laboratory of Pollution Processes and Environmental Criteria (Ministry of Education), Tianjin Key Laboratory of Environmental Remediation and Pollution Control, College of Environmental Science and Engineering, Nankai University, Tianjin 300071, China

## Abstract

In order to understand the composition and dynamics of planktonic viruses and their relationship with environmental parameters in natural freshwater, flow cytometry was optimized with filtration/fixation/staining/dilution and then applied to the analysis of samples collected from 9 stations (covering urban, rural, and estuarial areas) along the Haihe River, China, over a one-year period of study. The total viral abundance exhibited an apparent peak in the spring. Spatially, the highest viral abundance was recorded in estuarial areas. The correlation analysis indicated that the bacteria in the Haihe River significantly influenced viral abundance. The relationship between abiotic variables and viral abundance remained the same as with bacterial abundance, indicating that environmental parameters could possibly influence viral abundance in virtue of their bacterial host cells. The influence of environmental factors on viral abundance differed in the three sampling areas, suggesting different drivers of viral abundance in different stretches of the river associated with their utilization and surroundings.

## 1. Introduction

Over the last two decades, it has been realized that viruses are the most abundant biological entities in aquatic ecosystems (10^5^–10^8^ mL^−1^) and an important component in the aquatic microbial food web [1–4]. Viruses play critical roles in shaping aquatic communities and determining ecosystem dynamics and have been shown to affect nutrient cycling [5], microbial and dimethylsulfide release [6], and genetic material transfer [7]. Early studies of the roles of planktonic viruses were mainly focused on the marine pelagic environment, and more recently on other aquatic habitats such as lakes and rivers [8–10]. As a consequence, the roles of freshwater virioplankton remain much less studied. Heterotrophic bacteria and/or chlorophyll-a have often been reported to correlate with viral abundance [11–14]. Moreover, environmental parameters have also been observed to influence viral abundance [15–18]. The viral abundance and dynamics in aquatic environments can be influenced not only by biotic factors, but also by the combined effects of biotic and abiotic factors.

Easy and precise assays for the rapid counting of viruses are crucial for studies of viral ecology. Viruses in natural samples are traditionally enumerated by culturing methods and microscopic techniques such as transmission electron microscopy (TEM) [1, 19–22] and epifluorescence microscopy (EFM) [22–24]. Since the first report by Marie and coworkers [25], viruses in natural samples have been counted by flow cytometry (FCM) in numerous studies [15, 26–31]. While Brussaard and colleagues [32] provided a detailed discussion of FCM procedures for virus detection, this area remains partly unexplored as previous studies were predominantly focused on marine ecosystems. Because of the notably different properties of seawater and freshwater and their different microbial communities, it is worth of discussing the effects of the protocol in freshwater ecosystems. 

The aims of the current study were (1) to detect and quantify free viruses in natural freshwater using FCM combined with optimization of the filtration/fixation/staining/dilution parameters, (2) to analyze the seasonal and spatial changes in viral abundance along the Haihe River, China, using the optimized FCM method, and (3) to compare the influence of biotic and abiotic factors on viral abundance in freshwater.

## 2. Materials and Methods

### 2.1. Sampling Location

**Figure 1:**
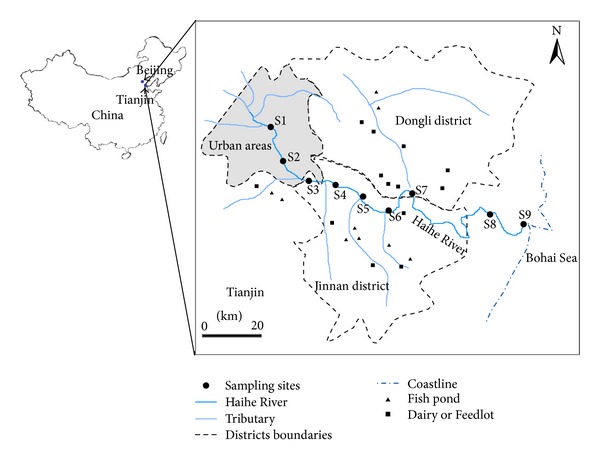
Location of sampling sites along the Haihe River, China.

Natural freshwater samples for different tests were collected from the Haihe River, which is the largest water system in Northern China. Water samples were collected at 9 sites along the Haihe River during the autumn (September, 2011), winter (December, 2011), spring (April, 2012), and summer (July, 2012) (Figure 1). Sites M1 and M2 were located in urban areas. Sites from M3 to M7 were located in agriculturally influenced areas. Sites M8 and M9 were located near the mouth of the Haihe River at distances of 8 and 1 km, respectively, from the Bohai Sea. Water samples were collected in clean, 2 L sterile bottles from a depth of 0.5 m at each sampling sites. 

### 2.2. Treatments and FCM Analysis

Working solutions of the dyes were prepared as follows: SYBR Green I (10,000X in dimethyl sulfoxide (DMSO), Invitrogen) was diluted 100 times in 0.20 *µ*m-filtered DMSO. Propidium Iodide (30 mM in DMSO, Invitrogen) was mixed with SYBR Green I stock at a ratio of 1 : 50. 

The method for virus detection in freshwater was first optimized with aquatic samples from the Haihe River, and then the optimal protocol was applied to the investigation of viral abundance variation. The procedures used as a reference protocol were filtration of 10 mL water samples through sterile syringe filters with 0.22 *μ*m pore size (PES, Millipore, USA) followed by fixation with glutaraldehyde (0.25% final concentration) for 15 min at 4°C and freezing in liquid nitrogen during transport and storage at −80°C after arrival in the laboratory. Na_2_EDTA (5 mM) was added to samples immediately prior to staining, followed by incubation with 5 *μ*L mL^−1^ SYBR Green I (100X in DMSO; Molecular Probes) at 80°C for 10 min in the dark. Samples were diluted in Milli-Q water prior to FCM analysis. To optimize the protocol, individual parameters (i.e., filtration, fixation, EDTA addition, staining temperature, and dilution solution) were optimized while keeping the other parameter fixed.

For enumeration of bacterial abundance, water samples were put on ice during transport and storage at 4°C after arrival in the laboratory and analysed within 6 hours. Staining and FCM analysis of bacteria were performed as described by Berney and colleagues [33]. In short, samples (1 mL) were stained with 10 *µ*L mL^−1^ SYBR Green I/Propidium Iodide staining solution and incubated for 25 min in the dark at room temperature. Prior to flow cytometric analysis, water samples were diluted with Milli-Q water.

Flow-cytometric measurements were made using a Partec CyFlow Space flow cytometer (Partec GmbH, Münster, Germany) with 488 nm excitation from a blue solid-state laser at 50 mW. All samples were collected as logarithmic signals and were triggered on the green fluorescence. Data were acquired on two-parameter dot plots of green fluorescence (520 ± 20 nm) versus sideward scatter (SSC) for virus and green fluorescence (520 nm, FL1) versus red fluorescence (630 nm, FL3) for bacteria. The output data were analysed with the Flomax software.

### 2.3. Environmental Parameters

A YSI EC300 Water Quality Sonde was used to measure water temperature and salinity on location. Total suspended solids (TSS), total nitrogen (TN), nitrate (NO_3_–N), total phosphorus (TP), total dissolved phosphorus (TDP), total organic carbon (TOC), and chlorophyll a (chl a) were measured according to standard methods [34]. The rainfall data were acquired from the Tianjin water resources bulletin.

### 2.4. Statistical Analysis

Seasonal and spatial distribution differences in viral and bacterial abundance were tested with one-way analysis of variance (ANOVA). Potential relationships between all microbial and environmental data sets were tested by Spearman's rank correlation analysis. Statistical analysis was performed with the Statistical Product and Service Solutions (SPSS) software (version 13.0).

## 3. Results and Discussion

### 3.1. Optimization of Procedures for Enumeration of Freshwater Viruses by Flow Cytometry 

#### 3.1.1. Filtration

**Figure 2:**
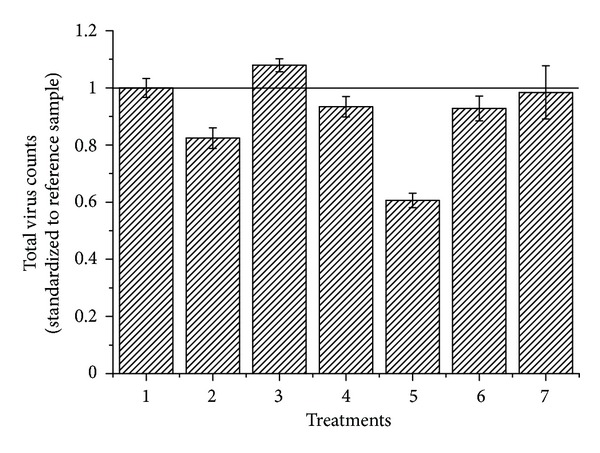
Comparison of total virus counts obtained by different treatments (1: reference; 2: filters with pore sizes of 0.1 *μ*m; 3: no filtration; 4: fixation with 0.5% glutaraldehyde; 5: staining at room temperature; 6: without EDTA; 7: diluted in TE). The total virus count of each treatment was normalized to samples tested under reference conditions. Error bars represent the standard deviation (*n* = 9).

**Figure 3:**
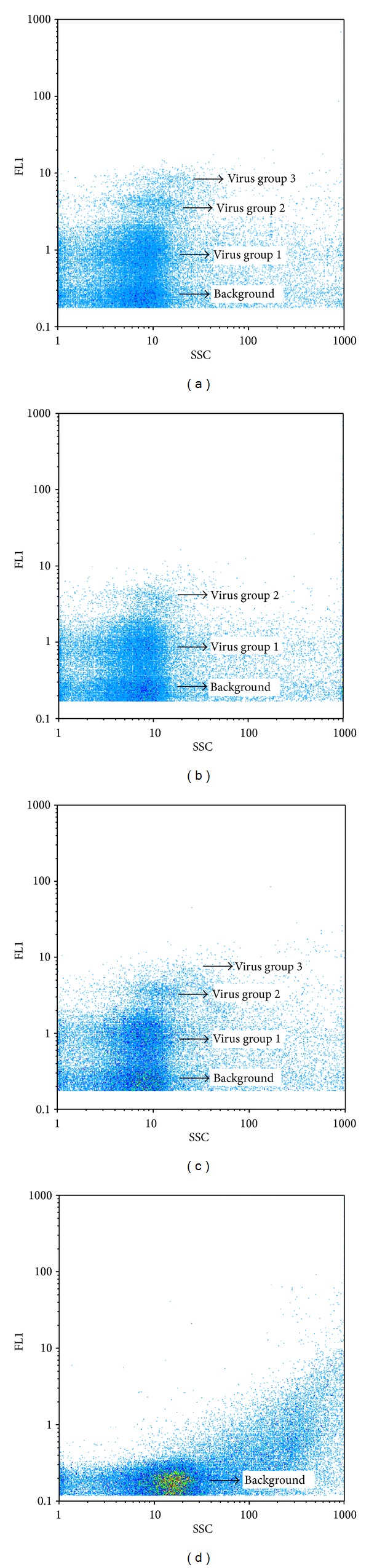
Flow cytometric analysis of viral populations by reference method (a), the same sample filtered through membrane filters with pore sizes 0.1 *μ*m (b), without EDTA (c), blank sample (using autoclaved 0.1 *μ*m pore-size prefiltered natural freshwater) analysed by reference method (d).

The total virus counts of samples passed through membrane filters with pore sizes of 0.22 *μ*m and 0.1 *μ*m were 7% and 24% lower than the counts of samples without filtration, respectively (Figure 2). However, this does not mean that filtration is negligible. Not every fluorescent dot might be a virus but could instead be DNA bound to colloids [11]. DNA fragments could be generated from bacterial cell lysate as a result of the treatment steps (e.g., freezing step). A filtration step prevents overestimation of the virus counts by bacterial lysate. When filtering through a 0.1 *μ*m pore size filter, it was evident that the natural virus communities lost a group of viruses compared to the sample filtered through a 0.22 *μ*m pore size filter and the unfiltered sample (Figures 3(a) and 3(b)). The total virus counts of the sample passed through the 0.22 *μ*m pore size filter were slightly decreased compared to the unfiltered sample, but none of the virus groups were missing. Based on these results, a good compromise choice would be to filter the sample through membrane filters with a pore size of 0.22 *μ*m, not only to reduce the bacteria and the influence of their cell lysates, but also to maintain reliable virus counts.

#### 3.1.2. Fixation

The loss of counts from fixed samples has been well documented. Similar to other reports [31], it was observed that the number of viruses in samples significantly decreases with the increased amount of glutaraldehyde (*P* < 0.05). This could result from glutaraldehyde being a potent virucidal agent. For bacteriophages, glutaraldehyde possibly forms protein-DNA cross-links, inhibiting DNA synthesis [35]. Instead of the optimized fixation concentration (0.5%) reported for marine viruses, we found that 0.25% glutaraldehyde gave a better fixation for freshwater viruses.

#### 3.1.3. Staining

EDTA was added to samples immediately before staining. Adding EDTA to samples not only positively influenced the total virus count, but also enhanced the FL1 and SSC signals compared to those of samples without EDTA (Figures 2 and 3(c)). In the present study there was a 39% reduction (*P* < 0.05) in the total virus count of samples stained at room temperature compared to samples heated at 80°C, which suggests that the high staining temperature is crucial to avoid underestimating the natural virioplanktonic abundance. This is consistent with a previous report that two of the tested marine heterotrophic phages showed a significant reduction in total virus counts at 60°C [31]. 

#### 3.1.4. Dilution

To avoid coincidence, the virus samples are normally diluted before loading onto a flow cytometer. However, the choice of diluting agent influences the measured virus concentration. To examine the effects of diluting agent, we compared autoclaved TE buffer through 0.1 *µ*m pore size membrane filters with dilution in Milli-Q water. It was observed that dilution in Milli-Q water provides a low instrument background and good results for discrimination of virus groups (Figure 3(a)). In addition, the quality of Tris may differ depending on the supplier, so it is likely to affect the quality of TE buffer and small batches of the TE buffer should be prefiltered immediately prior to use. It appears, therefore, that instead of TE buffer, dilution in Milli-Q water is more convenient and consistent.

#### 3.1.5. Detection Limit and Comparisons with EFM

**Figure 4:**
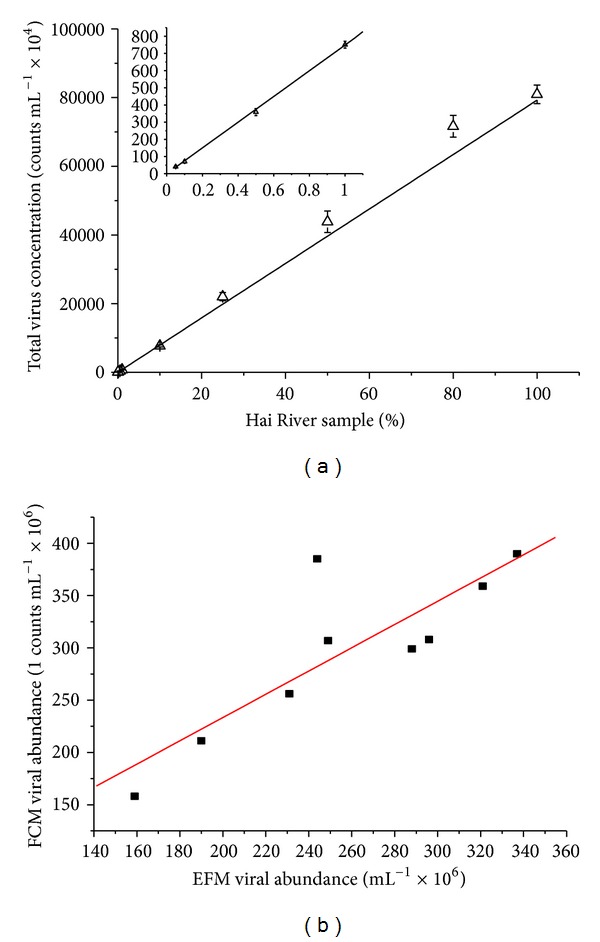
FCM detection limit and comparisons with EFM. Precision and detection limit of FCM for measuring total virus count (*n* = 9) (a); linear regression of viral abundance determined by FCM and EFM (FCM = 1.11EFM + 1.12, *r* = 0.84, *n* = 9) (b).

The precision and detection limit of FCM for measuring total virus counts was determined with a serial dilution of freshwater samples. Freshwater samples from the same sampling site in the Haihe River were serially diluted with Milli-Q water.  Figure 4(a) shows the detection limit of the instrument used in this study; *r* of the trend line is 0.99 (*n* = 9) and the lowest viral concentration detected is 4.04 × 10^4^ counts mL^−1^ (Figure 4(a)). Comparisons between FCM and EFM (using the protocol of Patel and coworkers [36]) for the enumeration of viruses were performed with 10 samples from the Haihe River. A good linear relationship was observed between FCM and EFM (*r* = 0.84, *n* = 9, Figure 4(b)). 

The results obtained in this study suggest that when studying the abundance of viruses in freshwater, samples should be filtered with a 0.22 *μ*m pore size membrane, fixed with glutaraldehyde (0.25% final concentration) for 15 min at 4°C, frozen in liquid nitrogen, and stored at −80°C. For FCM analysis, samples should be added with EDTA (5 mM) immediately before staining and incubated with 5 *μ*L mL^−1^ SYBR Green I (100X in DMSO) at 80°C for 10 min in the dark, then diluted in Milli-Q water prior to analysis.

### 3.2. Seasonal and Spatial Distribution of Bacterioplankton and Virioplankton

**Figure 5:**
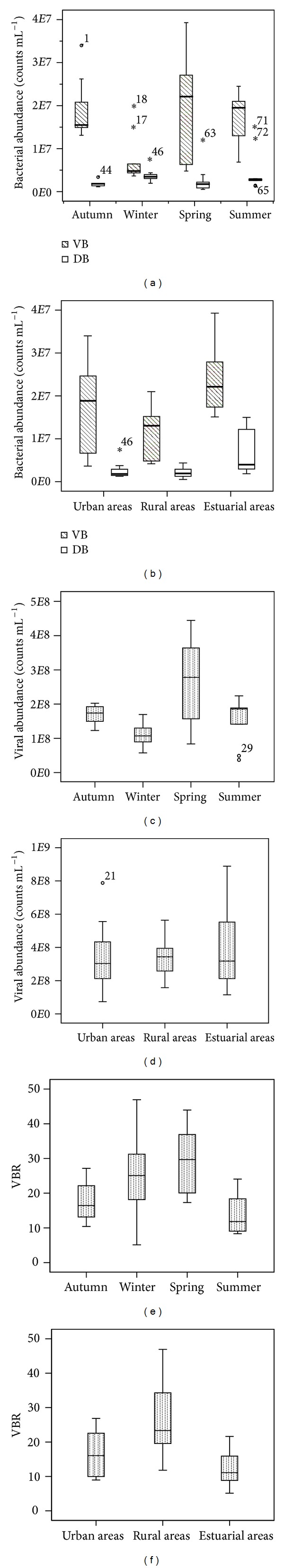
Boxplots of microbial abundance and VBR from samples collected along the Haihe River. (a) Seasonal distribution of bacterial abundance (VB, viable bacteria, *n* = 36; DB, dead bacteria, *n* = 36); (b) spatial distribution of bacterial abundance (VB, viable bacteria, *n* = 36; DB, dead bacteria, *n* = 36); (c) seasonal distribution of viral abundance (*n* = 36); (d) spatial distribution of viral abundance (*n* = 36); (e) seasonal distribution of VBR (*n* = 36); (f) spatial distribution of VBR (*n* = 36); outliers numbers 1 and 46 refer to site S1; 29 and 65 refer to site S2; 21 refers to site S3; 17, 44, and 71 refer to site S8; and 18, 63, and 72 refer to site S9.

Viable and dead bacteria were discriminated between on the basis of their membrane integrity using a combination of SYBR Green I and PI [33]. The total cell count is considered to be the sum of viable and dead cells. The abundance of viable bacteria showed a dramatic fluctuation ranging from 3.64 × 10^6^ to 3.93 × 10^7^ counts mL^−1^, with a clear seasonal pattern as evidenced by low abundance during the winter (Figure 5(a)). The optimized treatment method was applied to virus enumeration of samples collected at 9 sites along the Haihe River over a one-year period. Total viral abundance over the course of the investigation ranged from 7.35 × 10^7^ counts mL^−1^ to 8.88 × 10^8^ counts mL^−1^ (Figure 5(c)), which is consistent with the range reported by previously published studies on viral abundance values in freshwater environments such as rivers, ponds, and lakes [8, 10, 26].

Viral abundance in freshwater is reported to undergo stronger seasonal changes than those in marine environments, especially in lakes [9]. In this study, the viral abundance peaked in spring and reached its lowest level in winter (*P* < 0.01). The lowest values of viral concentrations observed in winter may be explained by the fact that the host bacteria were least abundant in the winter, and thus fewer viruses would be released into the water. In contrast to previous reports [37, 38], the highest viral abundance was recorded in the spring rather than in the summer (Figure 5(c)) in the present study. The reason may be due to the high intensities of solar radiation in the hot weather in northern China, which could accelerate viral degradation and viral decay [16, 17].

As shown in the boxplot (Figures 5(a) and 5(c)), several outliers were identified in sampling site 1, site 2, site 8, and site 9, which indicated that the spatial location potentially influenced the microbial abundance. Thus, according to the different environmental types of the sampling sites, microbial abundance was categorized into urban areas, rural areas, and estuarial areas (Figures 5(b) and 5(d)). It is notable that the average concentrations of viable and dead bacteria were both higher in estuarial areas, followed by urban areas, and lower in rural areas. The results from one-way ANOVA showed that the viable, dead bacterial abundance was significantly different between the three areas (*P* < 0.01). Although no significant spatial changes in viral abundance were observed, the average abundance of virus increased from 3.01 × 10^8^ counts mL^−1^ in urban areas to 3.49 × 10^8^ counts mL^−1^ in rural areas and continuously went up to 3.96 × 10^8^ counts mL^−1^ in estuarial areas. 

The virus-to-bacteria ratio (VBR), indicating the relationship between viral and bacterial communities, exhibited a clear seasonal and spatial variation in the study (Figures 5(e) and 5(f)). The VBR fluctuated from 5.12 to 46.94 with an overall mean of 21.44, which demonstrated the numerical predominance of viruses over bacteria, consistent with previous reports [2]. The VBR values were significantly higher in the spring and lower in the summer (*P* < 0.01) although the bacterial abundance in the two seasons did not significantly change. This indicates that there are possible factors other than the host influencing the dynamics of viral abundance in the study area. The VBR values were significantly different among urban, rural, and estuarial areas (*P* < 0.01), indicating the inconsistency of virus-host interactions between the three areas.

### 3.3. Correlation Analysis

The nutrient concentrations (TN, NO_3_–N, TP, and TDP), physical parameters (temperature, salinity, and TSS), TOC, and chl a of each site are shown in Table 1. Rainfall displayed a significant seasonal variability with 17.02 mm in the spring, 97.29 mm in the summer, 60.53 mm in the autumn, and 5.81 mm in the winter. In this study, correlation analysis was used to identify physical, chemical, and biological variables that are associated with changes in viral abundance. 

#### 3.3.1. Biotic Influence on Viruses in the Haihe River

Previous reports have demonstrated that high viral abundance is typically associated with high bacterial abundance and/or chlorophyll-a concentration [10, 13, 15, 39, 40]. Maranger and Bird reported that a strong correlation was found between viral abundance and chlorophyll-a concentration, but not with bacterial abundance in lakes, whereas viral abundance in marine systems was strongly correlated with bacterial abundance [12]. However, in our study, significant relationships between viral abundance and chlorophyll-a concentration (0.440, *P* < 0.01) and between viral and bacterial abundance (0.575, *P* < 0.01) were found from analysis of data for the 9 sites. As shown in Table 2, when the data were analyzed in separate regions, the relationships between viral abundance and chlorophyll-a concentration became weak, but the bacterial abundance still strongly influenced the viral abundance.

#### 3.3.2. Effects of Environmental Parameters on Viral Abundance

Overall, no significant correlation was found between virioplanktonic abundance and abiotic variables over the whole river. However, when the data were analysed separately by different sampling regions, it was observed that there were several factors (rainfall, TN, temperature, salinity) in addition to the host that directly or indirectly possibly influenced the dynamics of viral abundance. We recognized that viral abundance was significantly negatively correlated with salinity in rural areas (*r* = −0.602, *P* < 0.01), supporting a previous hypothesis [38], which could be due to the changed ionic strength affecting the viral replication ability and even absorption to particles [2, 12]. However, viral abundance was positively influenced by salinity in estuarial areas (both *r* = 0.69 and *P* = 0.05), indicating the different roles of salinity in the two areas. The correlation between bacterial abundance and salinity was also negative in the rural areas but positive in estuarial areas (Table 2), which indicates that salinity may indirectly affect viral abundance via their hosts. Otherwise, the opposite effects of salinity in the two areas suggest that the predominant bacterial and viral communities were possibly changed between rural and estuarial areas and that the upstream production of native microbes could not contribute to the abrupt growth of microbes in estuarial areas. The rainfall pattern over the Haihe River is strongly seasonal with wet summers and low winter rainfall. We found that the amount of rainfall positively influenced the viral (*P* < 0.05) and bacterial abundances (*P* < 0.01) in rural areas (Table 2). Such correlations have been reported before, with seasonal differences in viral and bacterial abundance and virus-host interactions being greatly influenced by rainfall [41]. During this study, we found that the amount of rainfall in rural areas greatly affected the measured environmental parameters. For instance, we observed a positive correlation with water temperature (*r* = 0.753, *P* < 0.001), TP (*r* = 0.617, *P* < 0.01; often regarded as nonpoint pollutions in agricultural areas), TDP (*r* = 0.586, *P* < 0.01) and chl a (*r* = 0.574, *P* < 0.01), and a negative correlation with salinity (*r* = −0.523, *P* < 0.05). Thus, the positive impact of rainfall on microbial abundance could be explained by the significant input of nutrients from the surface runoff, with phosphorus from the soil flowing into water bodies. Meanwhile, the salinity could also be diluted, thus promoting growth of host bacteria in this region and leading to increased virus counts. During our sampling period, bacterial and viral abundance was significantly and positively correlated with temperature in rural areas, supporting the idea that temperature is an important environmental factor controlling microbial growth [9]. Temperature may play an indirect role in viral abundance, as high temperatures benefit bacteria growth resulting in a higher number of hosts for bacteriophages. Moreover, higher numbers of viruses were associated with elevated nutrient concentrations (TN and TP) in estuarial areas (Table 2). Such a positive correlation has also been documented in other water bodies [9, 42–44]. Some of these patterns probably result from the promotion of bacterial productivity under eutrophic conditions, as well as the encouraged growth of bacterial populations that might serve as viral hosts. 

These results show that viral and bacterial abundance was both influenced by environmental factors. The level of correlation between abiotic variables and viral abundance was always similar to that of the relationship between abiotic variables and bacterial abundance (Table 2), indicating the importance of indirect influence on viruses via their bacterial host cells. The environmental factors influencing viral abundance varied in the three sampling areas, suggesting that the viral abundance in different stretches of the river with their specific surroundings was possibly driven directly and indirectly by different abiotic factors.

## 4. Conclusions

1. The present study has provided an adequate and fast method using flow cytometry to investigate the variation of viral abundance in freshwater environments.
2. Viral distribution could be influenced both by biotic and abiotic variables.
3. The effects of abiotic variables on viral abundance may also lie in the indirect influence via their bacterial host cells.
4. There were clear differences in microbial abundance among the three sampling environments (urban, rural, and estuarial areas), and the abiotic influence on viral abundance was different in all three environments.

## Figures and Tables

**Table 1:** Environmental data recorded for the Haihe River from September 2011 to June 2012.

	Autumn		Winter		Spring		Summer	
	Mean (min–max)	Median (Q1, Q3)	Mean (min–max)	Median (Q1, Q3)	Mean (min–max)	Median (Q1, Q3)	Mean (min–max)	Median (Q1, Q3)
Temp (°C)	28.2 (27.6–29.4)	28.2 (27.8, 28.5)	12.6 (11.0–15.6)	12 (11.7, 13.6)	13.4 (12.5–14.2)	13.3 (24.9, 25.3)	25.0 (24.0–25.7)	24.9 (11.7, 13.6)
TSS (mg L^−1^)	5.59 (3.12–9.68)	5.00 (4.06, 6.25)	8.19 (0.31–15.62)	7.50 (5.00, 13.43)	8.73 (0.31–21.77)	5.62 (10.62, 31.33)	31.85 (4.06–100.00)	20.66 (5.00, 13.43)
TN (mg L^−1^)	4.68 (3.71–5.41)	4.80 (4.49, 4.98)	4.50 (3.04–5.20)	4.65 (4.27, 5.10)	7.79 (4.33–10.14)	8.76 (6.23, 6.33)	6.25 (6.00–6.43)	6.23 (4.27, 5.10)
Nitrate (mg L^−1^)	2.14 (1.63–2.41)	2.16 (2.03, 2.35)	2.44 (2.06–2.85)	2.39 (2.36, 2.48)	2.08 (1.26–2.87)	2.09 (0.88, 2.02)	1.53 (0.82–2.90)	1.50 (2.36, 2.48)
Chla (*μ*g L^−1^)	107.61 (84.40–156.00)	107.00 (86.2, 116)	15.97 (4.00–47.00)	13.00 (9.30, 17.80)	62.67 (6.63–236.35)	25.72 (34.35, 132.41)	91.62 (3.76–262.31)	69.95 (9.30, 17.80)
TP (mg L^−1^)	0.76 (0.60–0.99)	0.74 (0.74, 0.77)	0.42 (0.34–0.54)	0.39 (0.36, 0.48)	1.96 (0.27–8.08)	0.55 (0.58, 1.09)	0.82 (0.14–1.26)	0.96 (0.36, 0.48)
TDP (mg L^−1^)	0.47 (0.37–0.56)	0.47 (0.45, 0.51)	0.26 (0.17–0.44)	0.24 (0.18, 0.31)	0.34 (0.12–0.69)	0.24 (0.21, 0.58)	0.45 (0.10–0.71)	0.55 (0.18, 0.31)
TOC (mg L^−1^)	17.14 (0.10–53.93)	10.77 (9.55, 13.9)	30.80 (16.71–41.13)	31.26 (25.39, 36.80)	22.05 (6.31–68.78)	16.97 (18.30, 26.76)	20.54 (5.84–28.86)	21.85 (25.39, 36.80)
Salinity (‰)	2.6 (1–6)	2 (2, 2)	3.9 (1–9)	3 (2, 3)	5.2 (1–18)	2 (2, 2)	4.7 (1–20)	2 (3, 3)

Q1: 1st quartile; Q3: 3rd quartile; Temp: temperature; TSS: total suspended solid; TN: total nitrogen; chl a: chlorophyll a; TP: Total phosphorus; TDP: total dissolved phosphorus.

**Table 2:** Correlation matrix (*r*) of biotic and abiotic parameters in the Haihe River.

	Temp	TSS	TN	Nitrate	Chl a	TP	TDP	Rainfall	TOC	Salinity	DB	VB	TV	TB
Whole river														
DB	−0.304	0.262	−0.156	0.191	0.025	0.005	−0.148	−0.175	0.362	0.530	1.000	0.146	0.105	0.387*
VB	0.323	0.106	0.164	−0.214	0.514**	0.171	−0.004	0.409*	−0.117	0.126	0.146	1.000	0.575**	0.946**
TV	0.169	−0.085	0.177	0.025	0.440**	0.189	0.064	0.220	−0.227	−0.177	0.105	0.575**	1.000	0.568**
TB	0.285	0.116	0.033	−0.186	0.489**	0.178	0.032	0.380*	−0.049	0.232	0.387*	0.946**	0.568**	1.000
Urban areas														
DB	−0.595	−0.671	−0.476	0.619	−0.119	0.429	0.381	−0.927**	0.381	0.591	1.000	−0.190	0.524	0.286
VB	0.167	0.299	0.119	0.024	0.619	0.167	−0.048	0.293	−0.214	−0.206	−0.190	1.000	0.619	0.833*
TV	−0.500	−0.455	−0.381	0.619	0.286	0.286	0.071	−0.488	−0.190	−0.027	0.524	0.619	1.000	0.881**
TB	−0.095	−0.096	−0.333	0.262	0.643	0.429	0.190	−0.146	−0.095	−0.014	0.286	0.833*	0.881**	1.000
Rural areas														
DB	−0.294	0.322	−0.423	0.391	0.019	−0.362	−0.524*	−0.124	0.444	0.131	1.000	−0.090	−0.086	0.180
VB	0.680**	0.151	0.127	−0.494*	0.587**	0.202	0.229	0.760**	−0.203	−0.477*	−0.090	1.000	0.651**	0.937**
TV	0.461*	−0.201	0.191	−0.234	0.432	0.088	0.103	0.543*	−0.126	−0.602**	−0.086	0.651**	1.000	0.633**
TB	0.618**	0.137	−0.104	−0.357	0.594**	0.043	0.068	0.721**	−0.144	−0.498*	0.180	0.937**	0.633**	1.000
Estuarial areas														
DB	−0.071	0.786*	0.667	−0.238	0.048	0.333	0.228	0.439	0.119	0.667	1.000	0.286	0.548	0.714
VB	0.024	0.286	0.571	0.262	0.690	0.143	−0.575	−0.049	0.000	0.357	0.286	1.000	0.690	0.833*
TV	0.190	0.286	0.762*	0.024	0.667	0.619	−0.204	0.390	−0.167	0.690	0.548	0.690	1.000	0.833*
TB	0.048	0.643	0.810*	−0.095	0.619	0.357	−0.228	0.293	0.000	0.690	0.714*	0.833*	0.833*	1.000

*P < 0.05; **P < 0.01.

DB: dead bacteria; VB: viable bacteria; TV: total viral abundance; TB: total bacteria abundance.
